# AI Dermatology's Unfulfilled Promise: Why Early Melanoma Detection Remains Unverified

**DOI:** 10.1111/ijd.70636

**Published:** 2026-09-29

**Authors:** Tim J. Zeuner, Titus J. Brinker

**Affiliations:** ^1^ Division of Digital Prevention, Diagnostics and Therapy Guidance German Cancer Research Center (DKFZ) Heidelberg Germany; ^2^ Medical Faculty University Heidelberg Heidelberg Germany

**Keywords:** artificial intelligence, Breslow thickness, dermoscopy, diagnostic uncertainty, early detection, explainable AI, histopathology, longitudinal imaging, machine learning, melanoma

## Abstract

A growing number of artificial intelligence (AI) systems for melanoma diagnosis now match dermatologist‐level performance. Early detection is a driving factor, yet it is rarely validated beyond aggregate diagnostic accuracy. More targeted studies are necessary so these claims become verifiable and the promise of early detection can actually be delivered to patient care.

Early detection is cited as a primary motivation in nearly all publications on artificial intelligence (AI)‐assisted melanoma diagnosis. The rationale is compelling: survival rates drop substantially with increasing Breslow thickness, and detecting melanoma at its earliest stages remains the most effective way to reduce melanoma mortality. AI systems have demonstrated diagnostic performance comparable to that of dermatologists in prospective clinical studies [1]. To improve patient care, however, integrating artificial intelligence into dermatology involves more than maximizing sensitivity and specificity. Available studies provide little insight into how models perform across clinically relevant strata such as Breslow thickness or stage, and remain too diagnostically focused to establish clinical utility.

The challenge of early melanoma detection extends beyond dermatoscopy and into the current histopathological reference standard. Haggenmüller et al. [2] assessed interrater agreement among international expert pathologists reviewing histopathological whole‐slide images, the highest‐quality diagnostic input available. Noninvasive and pT1 melanomas showed substantial diagnostic variability among expert pathologists, with moderate interrater agreement and frequent absence of majority consensus.

These are not peripheral edge cases: Thin and noninvasive melanomas represent the majority of detections in screening settings. If expert pathologists cannot reach a reliable consensus on early‐stage lesions, the implications for AI‐based models for melanoma detection are serious. These systems are trained and validated on labels derived from a diagnostic process that is uncertain itself, and operate on dermoscopic or clinical images that carry far less diagnostic information. AI may therefore reproduce or amplify contemporary diagnostic uncertainty, and the claimed performance in early‐stage melanoma detection warrants greater scrutiny than the current literature affords.

A recent benchmark underscored how aggregate accuracy may obscure early‐stage failure. Most public datasets lack Breslow thickness or T‐category labels, and ISIC challenge‐winning models performed substantially worse on a benchmark enriched for in situ and thin melanomas than on standard ISIC datasets [3]. More clinically oriented evaluations are beginning to move beyond single‐timepoint accuracy. PanDerm assessed sequential images of 89 melanomas and detected suspicious change at the first imaging time point in 77.5% of sequences, compared with 32.6% for clinicians [4]. This represents an important step toward evaluating AI systems in a manner that more closely reflects clinical reality and remains one of the few approaches that explicitly address early detection. While such metrics are inherently threshold‐sensitive and do not fully capture specificity trade‐offs, they provide a valuable framework for moving beyond static accuracy measures.

Yet even stage‐stratified or longitudinal validation addresses only part of the evidence gap. Early detection remains essential for clinically consequential melanoma, but the value of detecting every additional early lesion is not self‐evident. Increased melanoma incidence, particularly for in situ and thin invasive melanomas, has often occurred without corresponding increases in mortality, raising concern that some of this increase reflects overdiagnosis rather than prevention of lethal disease [5]. For AI‐assisted diagnosis, this creates central clinical tension. Sensitivity‐optimized systems will improve early detection of consequential melanomas, but may also increase detection of indolent lesions that would not have become clinically relevant during a patient's lifetime.

To move beyond AI‐driven early melanoma detection as a recurring headline, future work must align more closely with the realities of diagnostic uncertainty and clinical decision‐making [6]. Against the background of effective immunotherapy and targeted therapy, the incremental benefit of detecting ever‐earlier lesions should be demonstrated rather than assumed. Future studies should combine stage‐stratified validation, especially for in situ and thin melanomas, with longitudinal datasets and clinically meaningful endpoints such as melanoma‐specific mortality, unnecessary procedures, or surveillance burden. Without such evidence, continued improvements in aggregate diagnostic accuracy risk overstating progress while leaving the central promise of AI‐driven early detection unfulfilled.

## Funding

The authors have nothing to report.

## Conflicts of Interest

T.J.B. is the owner of Smart Health Heidelberg GmbH, a software company which developed the first teledermatology app in Germany (“AppDoc” https://appdoc.de) and received honoraria from Novartis, Roche, Merck and HEINE Optotechnik. The other authors declare no competing interests.

## Data Availability

Data sharing is not applicable to this article as no datasets were generated or analysed during the current study.
